# Facetime vs. Screentime: Gaze Patterns to Live and Video Social Stimuli in Adolescents with ASD

**DOI:** 10.1038/s41598-019-49039-7

**Published:** 2019-09-02

**Authors:** R. B. Grossman, E. Zane, J. Mertens, T. Mitchell

**Affiliations:** 1 Emerson College, Boston, USA; 2 Brandeis University, Waltham, USA

**Keywords:** Psychology, Human behaviour

## Abstract

Atypical eye gaze to social stimuli is one of the most frequently reported and studied social behaviors affected by autism spectrum disorder (ASD). The vast majority of this literature is based on analyses of gaze patterns as participants view social information, such as talking faces, on a computer screen. However, recent results suggest that generalizing gaze behaviors from computer screens to live interactions may not be valid. This study examines between- and within-group differences in gaze behaviors of children with ASD and their neurotypical (NT) peers during a screen-based and a live-interaction task. Results show between-group differences in gaze only for the screen-based, but not the live-interaction task. We also find that gaze behavior of NT children during the screen-based task significantly correlates with their gaze behavior during the live interaction; individuals who direct a higher percentage of gaze to the face in one task also did so in the other task. However, there is no significant relationship between the gaze patterns of children with ASD for those two tasks. These results strongly caution against using gaze of individuals with ASD recorded during screen-based tasks as a proxy for understanding their gaze behavior during live social interactions.

## Introduction

One of the earliest and most consistent reports about individuals with Autism Spectrum Disorder (ASD) is the presence of atypical eye gaze to social stimuli, particularly faces. In the first clinical description of ASD, Kanner^1^ repeatedly mentions that his patients do not look at people near them, even during social interactions. Asperger^2^ describes children with higher verbal skills than those studied by Kanner, but his observations of gaze avoidance are very similar. Today, we still recognize atypical gaze to social information as a central manifestation of ASD, among other deficits in social reciprocity and restrictive and repetitive behaviors^3^ (DSM-5). For instance, the current gold standard diagnostic tool, the Autism Diagnostic Observation Schedule 2^nd^ edition^4^ (ADOS-2), includes atypical eye contact during social interactions as one target observation.

With the advent of eyetracking technology, researchers began turning their attention to interpreting more fine-grain features of gaze behavior. A formative paper by Klin *et al*.^5^ found that adults with ASD show decreased gaze to faces while watching video clips from *Who’s Afraid of Virginia Woolf*. Since then, methodologies and interpretation of eyetracking studies in ASD have become more nuanced and several studies describe autistic gaze patterns to faces that are not different from those of NT peers^6–8^. Recent meta-analyses and reviews of the eyetracking literature show that a majority of studies do find deficits in face-directed and particularly eye-directed gaze for individuals with ASD^9^. However, gaze patterns in this cohort are strongly modulated by the type of stimulus: static (photographs), dynamic (videos or interactive), social (e.g. images and videos showing people), or interactive. This is true whether participants are NT^10^ or autistic^11,12^. Differences in gaze patterns between diagnostic groups appear to be most pronounced in studies using stimuli with more social content^13,14^ or greater ecological validity, as defined by the authors^15^ (In the clinical literature, person-first language is preferred. However, many individuals with ASD prefer the term autistic adult/child. To reflect this dichotomy we use the two terminologies interchangeably).

The evidence that social content of a screen-based stimulus can have a significant impact on gaze data is particularly important, because eyetracking studies in ASD have almost exclusively used gaze to screen-based stimuli as a proxy for understanding interactive social gaze behavior in ASD. The recent literature in this area indicates that there at least two ways gaze behavior differs between screen-based social stimuli and live social interactions. First, data from NT adults indicates that gaze behavior recorded during a walk through the environment is different from gaze behavior in response to single, still images of the same environment^16^. Second, a recent review of related literature notes that people behave differently when looking at images of people as compared to people in their environment. For example, people make direct eye contact with images much more so than live social partners because they know that images are not returning their gaze^17^. The authors conclude that the high level of stimulus control afforded by lab-based studies does not and should not outweigh the interactive nature of live interactions and that social science research should move toward investigating live interactions, rather than using controlled lab-based studies^17^. Together, these data suggest that screen-based gaze behaviors may not be a valid proxy for understanding gaze behavior in interactive tasks.

The limited existing data on interactive eye gaze suggest that social responses to live interactions are likely different from those to carefully controlled screen-based social stimuli. These differences may be even more pronounced for individuals with ASD, due to the increased social stressors of direct, human-to-human eye gaze in this population^18,19^. For example, eye contact with a conversation partner during a live interaction significantly improved the ability of NT children to encode a sequence of random digits, but had no such effect on children with ASD^20^. Given these findings, the impact of face-to-face interaction on social behavior may be different for individuals who have a documented deficit in reciprocal social communication.

Previous studies have explored this relationship, by analyzing the impact of general social communication skills on gaze patterns. Using scores on the Autism Quotient^21^ (AQ) as a measure of social ability, Chen and Yoon^22^ found that NT adults with higher AQ scores (i.e. more autistic-like behaviors) looked more at speaking faces with averted eyes than with direct gaze in video clips, while participants with lower AQ scores looked more at faces with direct gaze. Importantly, levels of anxiety were not related to these gaze patterns, indicating that responses to direct vs. averted speaker gaze was not based on social anxiety, but on autism-specific features. Conversely, Freeth, Foulsham, and Kingstone^23^ studied eye gaze in 32 undergraduate students, and found that AQ scores were not related to gaze patterns. Instead, the conversation partner’s gaze direction (direct or averted) correlated with listener eye gaze, but only during live interaction. When participants viewed a video of a speaker using the same gaze directions and the same conversation topics as during the live interaction, there was no effect of speaker gaze on listener gaze. This pattern of results gives further credence to the idea that gaze responses to live vs. screen-based social stimuli cannot be easily equated. Finally, Vabalas and Freeth^24^ also studied NT undergraduates and found no effect of participant AQ scores or speaker gaze direction on the amount of face-directed gaze recorded during live interaction, although they did document that higher AQ scored led to reduced face exploration with shorter and less frequent saccades to the speaker’s face. Given the work of Buitelaar (1995^25^), it is not clear whether these gaze behaviors are motivated by social avoidance or a lack of social awareness. Overall, these data collectively demonstrate the complexity of untangling the relative contributions of speaker behavior, participant characteristics, interactional factors, and task design for our understanding of social gaze during live interactions.

It is also unclear whether these findings would extend to individuals with a confirmed diagnosis of ASD. Despite the fact that behavior during live social scenarios constitutes the ultimate test of ecological validity^12^, few studies measure social gaze in individuals with ASD during live interactions, presumably because the technology only recently developed to the point that reliable live eye tracking was possible. There are two studies of eye gaze in autistic adults during live, face-to-face interactions. One study used a head mounted tracker with hand coded gaze data and found that ASD and NT participants looked at the faces of their conversation partner for similar amounts of time. However, autistic participants gazed more at their conversation partner’s mouth, while NT participants gazed more at the eyes^26^. Hessels *et al*.^27^ employed a clever design using two remote eyetrackers and a dual mirror/camera system that allowed participants to maintain direct eye contact to live streams of each other’s faces while gaze was tracked from both participants. Participants were not given any instructions on how to interact during the five minutes of recording, so there were no constraints on speaking, listening, gaze, etc. Results show that pairs of adults with higher scores on the Autism Quotient (AQ) engaged in less reciprocal eye-directed gaze than pairs of adults with lower AQ scores.

There have been a few studies of live interactions focusing on gaze behaviors of preschool and early school-aged children with and without ASD. One study focused on young children with and without ASD who engaged with a conversation partner via Skype^28^. This allowed investigators to work with traditional, remote eyetracking of gaze to a screen and still use an interactive, live social scenario. The study showed that children with ASD, particularly those with higher severity and more limited communication skills, gazed more at the mouth than the eyes of the interaction partner when conversations were focused on discussing people’s emotions versus when they focused on factual descriptions. Two other studies^28,29^ used a WearCam to record gaze patterns and field-of-view video for young children engaged in several play scenarios with an adult, such as blowing bubbles and toy play. Findings show that children with ASD gaze like their NT peers when talking about “things people do”^28^ and when orienting to facial expressions, but are slower to orient to speech^29^, and produce less face-directed gaze with shorter fixations when talking about “things people feel”^28^. Using a head-mounted eyetracker, Hanley *et al*.^30^ showed that autistic children had overall less gaze to a conversation partner’s face than NT children or those with Specific Learning Impairment. Conversely, Nadig *et al*.^31^ found that school-aged children with ASD did not look at faces of conversation partners significantly less than NT controls. This discrepancy may stem from the fact that participants in the Hanley *et al*.^30^ study were mostly listening to their partner, while those in the Nadig *et al*.^31^ study were mostly speaking. Overall, the findings for this limited set of eyetracking studies of live interactions with autistic children suggest that gaze varies significantly with task demands. Some studies of children with ASD find no overall reduction in the amount of face-directed gaze^31^, some show significant face-gaze differences between the groups^30^, while others identify more complex patterns of subtle differences in the timing of gaze during face exploration^29,32^.

No previous study on gaze behaviors has used a within-participant design to determine whether gaze patterns to screen-based stimuli constitute a valid representation of gaze patterns in live interactions in this population. Freeth *et al*.^23^ recorded eye gaze during a pre-recorded, screen-based “conversation” and a live interaction, but different groups of people participated in the two tasks. During the live interaction, participants tended to look away from the face when eye contact was made, particularly when the participant was speaking, while no such effect was observed during the screen-based task. While this study provides valuable information about gaze differences across the two tasks, the use of two different participant samples limits the ability to conclude that the task elicits different behaviors in the same individuals.

Given that the vast majority of eyetracking studies in ASD use social stimuli that are non-dynamic (images), or dynamic (videos) but non-interactive, it is important to determine if gaze behavior during those types of stimuli credibly reflects gaze behavior during the types of interactions autistic individuals may encounter during daily life. Existing data has established that stimulus type has a significant impact on gaze patterns in ASD and that social interactions may represent a particularly stressful environment for these individuals that may affect their gaze patterns. However, there is little to no evidence on within-participant differences in gaze patterns to passive viewing vs. interactive social stimuli. We therefore aimed to investigate gaze using a design allowing for both within- and between-participant comparisons. We recorded eye gaze to faces using screen-based video stimuli as well as live interactions in adolescents with and without ASD. We hypothesized there would be a condition-by-group interaction, whereby autistic individuals would show relatively less gaze to the face than their NT peers during the social interaction task, but not in the passive-viewing task. The social nature of the live interaction should exacerbate gaze avoidance for individuals with ASD, while the passive-viewing task should not. Further, during the passive-viewing task, participants watch a single speaking face on an empty, black background. This is a salient visual target without distractors, which may promote similar visual exploration across both groups. Finally, we hypothesize that, if modulation of social content significantly affects gaze, then gaze patterns in the screen-based task will not be predictive of gaze in the live interaction.

## Results

We used SensoMotoric Instruments (SMI™) software to draw a dynamic area of interest (AOI) on the face of each adolescent in the screen-based stimulus videos for the entire duration of their respective one-minute narratives. Each AOI was a polygon that included only the face, but not the hair or ears. We adjusted the AOIs frame-by-frame to account for movement of the faces during the videos. All subsequent statistical analyses of the screen-based task are based on face-directed dwell time, defined as all gaze (fixations, saccades, etc.) to the face AOI as a percentage of the total length of the video. The shape of the AOIs changed dynamically throughout the video to conform to the height and width of the face on the screen, which varied depending on the speaker’s precise distance to the camera and angle. The average coverage of the face AOI was 9.9% of the screen across the four stimulus videos. Since all participants saw the same three videos, AOI coverage did not differ between groups.

For the interactive task, we also used the SMI software to define the face AOI on the webcam video of the RA recorded during each conversation. We used the same standards of creating a dynamic face AOI as during the screen-based task with a tight fit around the face. Similar to the screen-based task, the AOI shapes and sizes were dynamically fitted to each RA’s face throughout each interaction and the average coverage of the face AOI was 14.72% of the video camera image for ASD participants (min: 2.35%, max: 28.4%) and 11% of the camera image size for TD participants (min: 3.9%, max: 32.6%) across all interactions, with no significant group differences (*p* = 0.15). All subsequent statistical analyses of the live interaction are based on dwell time to the RA’s face AOI as a percentage of the length of the conversation. Since all measures reported for both tasks are dwell time as a percentage of stimulus availability, i.e. the length of the narrative videos and the length of each participant’s interaction, the differences in overall duration of the interactions should not significantly affect the resulting data.

**Figure 1:**
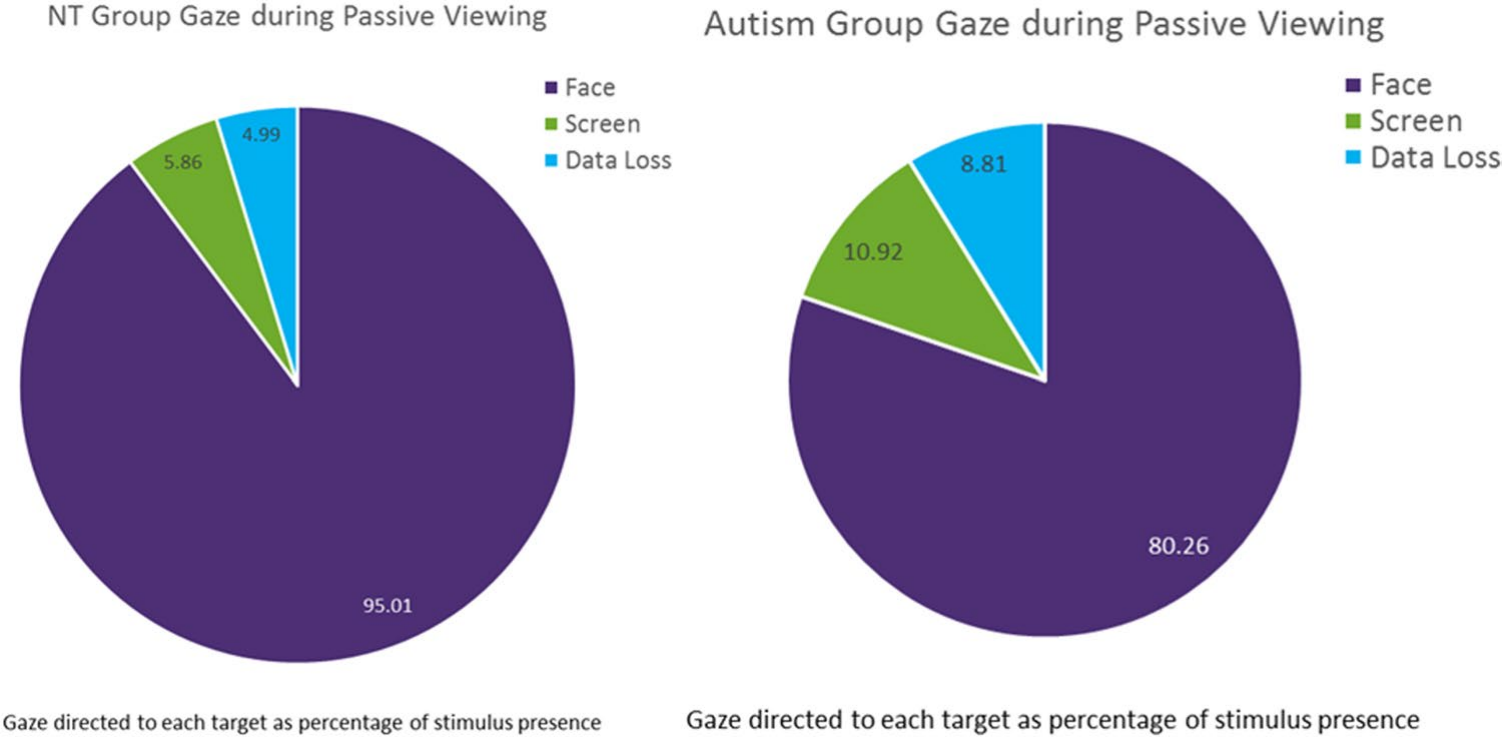
Gaze patterns during screen-based passive viewing.

We predicted that the ASD group would gaze less at faces than the NT group during the live interaction, but not the screen-based task. To test the hypothesis related to the screen-based task, we conducted a one-way ANOVA with diagnosis as the between-group variable and gaze to faces, gaze to the background (the entire screen without the face), and data loss (based on tracking ratio) during the passive viewing task as the test variables (Fig. 1). Results show significant differences in gaze to faces (*F* (1,30) = 5.0, *p* = 0.033, partial η^2^ = 0.15), with the ASD group gazing less at the face than the NT group (ASD: M = 80.26 StDev = 13.67, NT: M = 89.16 StDev = 89.16), as well as a significant difference in gaze to the background (*F* (1,30) = 5.63, *p* = 0.024, partial η^2^ = 0.16), with the ASD group gazing more at the background than the NT group (ASD: M = 10.9 StDev: 7, NT: M = 5.9 StDev = 5.86). There was no significant difference in the amount of data loss between the two groups (*F* (1,30) = 2.44, *p* = 0.13, partial η^2^ = 0.08, ASD: M = 8.81 StDev: 8.26, NT: M = 5, 5.41).

To test the hypothesis related to the live interaction task, we conducted the same analysis for gaze to faces and gaze to the background. Results show that there is no significant group difference in dwell time to the face (*F* (1,30) = 1.22 *p* = 0.28, partial η^2^ = 0.04, ASD: M = 21.5, StDev: 21, NT: M = 29.26, StDev: 17.71) or the background (*F* (1,30) = 0.5, *p* = 0.82, partial η^2^ = 0.002, ASD: M = 26.47, StDev: 17.53, NT: M = 27.88, StDev: 16.44). We did not conduct a group comparison of data loss, since the interactional nature of the task did not allow for that variable to be exported in a meaningful way. In the screen-based task, the stimulus and AOI presence were time-locked to data acquisition. However, in the live interaction task, data acquisition started prior to the task and included calibration, task instructions, the RA sitting down, etc. We therefore could not calculate the amount of data that was lost during the asking and answering portions of the live interaction task for which we present AOI statistics.

**Figure 2:**
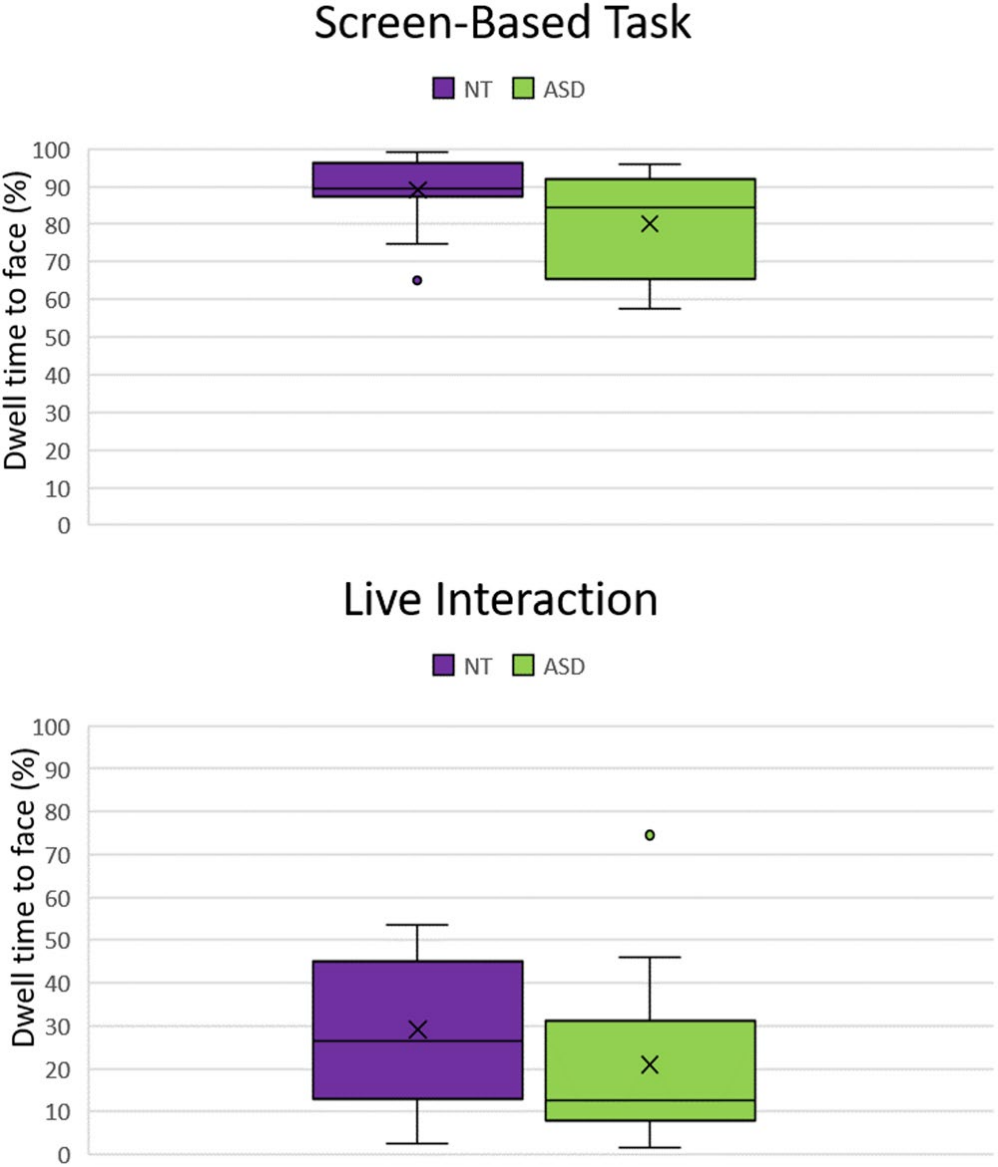
Face-directed gaze.

To compare gaze across both tasks we conducted a 2 (group) by 2 (task: screen-based vs. live interaction) mixed repeated measures ANOVA. The assumption for sphericity was met. Results show a main effect for condition (*F* (1,29) = 300.1, *p* < 0.0001, partial η^2^ = 0.91, observed power 1.0), with both participant cohorts gazing significantly more at faces during the screen-based task than the live interaction. There is a non-significant trend for NT participants to gaze more at faces than the ASD group (*F* (1,29) = 3.5, *p* = 0.07, partial η^2^ = 0.11, observed power = 0.44). There is no significant group by condition interaction (*F* (1, 29) = 0.01, *p* = 0.92, partial η^2^ < 0.0001, Fig. 2).

**Figure 3:**
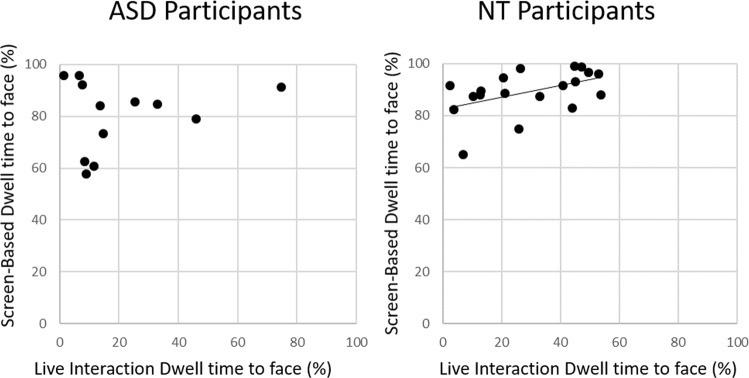
Scatterplots of gaze during screen-based and live interaction tasks.

We also hypothesized that gaze in the screen-based task would not predict gaze in the live interaction task. To test this hypothesis, we conducted separate linear regressions for the NT and ASD groups with dwell percentage during the screen-based task as the predictor and dwell percentage during live interaction as the dependent variable. Results show a significant relationship in gaze behavior across tasks for the NT participants, where dwell percentage during the screen-based task predicts 22% of the variance in dwell percentage during the live interaction (*F* (1,18) = 4.8, *p* = 0.04, *r* = 0.47, 95% CI [0.038, 1.92]). There is no significant relationship in face-directed gaze between the two tasks for the ASD group, with dwell percentage during the screen-based task predicting only 4% of the variance in dwell percentage during the live interaction (*F* (1,11) = 0.42, *p* = 0.53, *r* = 0.2, 95% CI [−0.76, 1.38], Fig. 3).

## Discussion

We present data on social gaze during a screen-based passive viewing task and a live interaction for autistic and NT adolescents. We first hypothesized that adolescents with ASD would have reduced gaze to faces compared to their NT peers in the live interaction but comparable face-directed gaze during the screen-based task. The data do not bear this hypothesis out. In the overall analysis of dwell percentage across both tasks, there was only a non-significant trend for NT participants to gaze more at faces than their autistic peers. When looking at each task separately, we found that this trend was driven by a significant reduction in gaze to faces by adolescents with ASD compared to their NT peers in the screen-based task. There was no significant difference in gaze patterns between groups during the live interaction. Contrary to our prediction, the group difference for face-directed gaze was only significant in the screen-based task and not the live interaction task. It is possible that the small sample size and relatively larger variance in the interactive task masked an underlying group difference. Future studies should use larger sample sizes to further explore this effect.

Our second hypothesis was that gaze patterns to the screen-based task would not predict gaze patterns during the live interaction. The data do support this hypothesis for the ASD but not the NT group, indicating that we cannot easily generalize social gaze from screen-based tasks to live interactions for adolescents with ASD.

The finding that both groups gazed significantly more at faces during the screen-based than the interactive task is not surprising. There is no social rule against staring at the face of a person represented on a screen, a behavior that may not be completely under volitional control^33^. It is therefore appropriate that both cohorts in our study gazed at the face during the screen-based task more than 80% of the time, on average. However, this tolerance for unlimited eye contact is not true for live conversation. According to long-standing data on typical conversational gaze behavior, we allocate direct gaze between 30% and 60% of time during live interactions^34^, which is in line with the significantly lower gaze percentages we found for the live interaction in both cohorts.

We predicted that the social pressures of direct face-to-face contact with an RA would reduce face-directed gaze in adolescents with ASD relative to the gaze patterns of their NT peers^22^. Our results do not support this hypothesis. Although our findings are somewhat surprising, they do resonate with data on younger children with ASD. A study of young children with and without ASD reported that, during a social engagement with an adult, the two groups did not differ in their gaze behavior toward the adult partner^35^. Similarly, Dawson *et al*.^36^ found no difference in gaze behaviors of toddlers and preschoolers with and without ASD during a variety of social interactions. In another study, García-Pérez *et al*.^37^ used an interviewing scenario similar to ours and coded partner-directed gaze behavior based on the resulting video recordings. Results showed that children with ASD did not look significantly less at their interviewer than NT children did. More recently, data on gaze patterns of 4–13 year olds coded from point-of-regard video glasses worn by an RA during toy play and non-toy conversation also found no difference between NT and ASD participants^38^. In that study, the frequency and duration of gaze directed at the partner’s face was significantly different based on context (toy play or conversation), but not different based on diagnosis for either condition. Collectively, these data from young children with ASD indicate that adult-initiated social conversations – at least the structured kind of interactions created in the laboratory - may elicit typical gaze patterns to partners faces.

There is also supporting evidence for our results in data from the general population. Freeth, Foulsham, and Kingstone^23^ found that AQ scores were negatively correlated with face-directed gaze in a cohort of undergraduate students. Higher AQ scores, which indicate more autistic behaviors, were correlated with reduced gaze to faces in a screen-based social viewing task. Importantly, this correlation between gaze behavior and autistic symptoms was not found in a separate live social interaction paradigm. In their study, as in ours, the difference between groups with high vs. low autistic traits was found for the screen-based task, not the live social interaction. Our study extends those findings in two important ways. First, our study included individuals with a verified diagnosis of ASD and an age-matched NT comparison group. Second, our design enabled within-participant statistical comparisons of eye gaze across live and screen-based tasks, while Freeth and colleagues^23^ conducted their two tasks with separate participant groups.

The most salient finding of our current study is the lack of a predictive relationship or significant correlation between gaze in a screen-based task and gaze during live interaction for the ASD group. It is possible that this lack of a cross-task correlation in the ASD cohort is in part related to the very high dwell time average and relatively low variance in the screen-based task that was found for both groups. Future research should consider this potential impact of near-continuous gaze to social stimuli on a screen that is not commonly found in live interactions. However, recent data from the general population do provide some foundation for this finding. Foulsham and Kingstone^16^ measured gaze of NT adults to static images taken from their environment and containing specific objects. When the same participants were later asked to walk through the same environment, filled with the same objects, the researchers found no predictive relationship of the gaze to objects in the static images and gaze to the same objects in real life. Our study differs from the work of Foulsham and Kingstone^16^ in that our two tasks involve dynamic social stimuli (speaking faces) presented against a simple and non-changing background. These differences in social content and dynamic properties may help explain why we found that NT participants’ gaze to screen-based stimuli does significantly predict gaze to the live, interactive face. Interestingly, the findings from our autistic group showing no significant relationship between gaze patterns across tasks, better match the findings reported in Foulsham and Kingstone^16^. This suggests that the social content and social demands of our interactive task may have affected the autistic participant in our study differently from their NT peers.

Previous work on gaze in interactive conditions show significant individual variation based on personality or social context^39^. Given the ubiquitous mentions of increased heterogeneity in the ASD population for most measures of social communication, these individual variations may come into play more significantly during social interaction and lead to a lack of relationship between screen-based and live social gaze in this cohort, but not in their NT peers. The majority of eyetracking studies in the ASD population continue to be screen-based. Our data indicate that there may be significant differences in viewing patterns for such passive viewing tasks compared to gaze behavior during live social interactions, particularly for adolescents with ASD.

## Limitations

This study focused on a relatively small cohort of adolescent ASD participants who did not have intellectual disability. Replication of our findings in a larger and broader sample of autistic individuals will be important, as well replication with tasks that allow for greater variability in gaze responses, rather than the near-continuous gaze found for the screen-based task. Replication with other technologies will also be important. Although it is within the capabilities of the SMI RED eyetracker to be used during live interactions, the ability of participants to move freely in their chairs and the greater amount of physical adjustments people make when talking compared to when they are watching a video, led to a greater-than-usual data loss. Future studies may consider using wearable eyetracking glasses, which allow for greater participant movement while still maintaining gaze tracking. More importantly, future studies should focus on interactive paradigms and consider interactional factors, such as times of listening vs. speaking, as well as behavioral responses of social partners.

As stated before, despite presenting participants with a single speaking face in both tasks, the two conditions were different from each other in many ways. We attempted to mitigate those differences by maintaining an identical physical setup, using the same conversation topics, and a within-subjects design. Participants were seated in the same position in both tasks, looked in the same direction, and completed the same calibration procedure for the eyetracker. However, the gaze of the RA^40^, presence and absence of verbal speech^29^, speaking time and listening time were uncontrolled. The absence of these controls limits our ability to investigate the contributions of these factors to the differences in gaze patterns we observed. Recent studies in the general population have begun using conversational tasks across different methodologies, such as video, Skype conversation, or a face-to-face live interaction, allowing for more direct comparisons of gaze behavior across task and social demands^41–43^. Such paradigms will allow us to also learn more about the specific factors that influence gaze behavior of autistic individuals during realistic conversational exchanges.

A further limitation of our study is that the live interactions revealed the diagnostic status and gender of each participant to the research assistants. This means that there may have been differences in the way RAs interacted with the participants in terms of their diagnosis or gender. We tried to mitigate these effects of observer bias by using consistent instructions and using several RAs in rotation.

## Conclusions

We find that the gaze patterns of autistic adolescents to dynamic social stimuli (i.e. a speaking face) presented on a screen are not predictive of gaze patterns to the face of a live communication partner. This suggests that we exercise caution in generalizing eye tracking results from screen-based tasks to live social interactions, at least for the autistic population. Future social communication eyetracking research in ASD should strive to investigate behaviors during live interactions.

## Method

For the screen-based tasks, we used passive viewing of a social video showing a young adult speaking and gazing directly into the camera to represent a high level of what has been referred to as ecological validity in a recent review of eyetracking studies^13^ and duplicate any potential effect of direct gaze^20^ present in the live interaction condition. We did not control gaze during conversation^40^, because our priority was to engage participants in a naturalistic social interaction. For both tasks participants were seated in same position, at the same table, and used the same eyetracker. The topics of the verbal interactions in both conditions were also identical to avoid the possible influence of conversational content area^28^.

### Screen-based task

**Figure 4:**
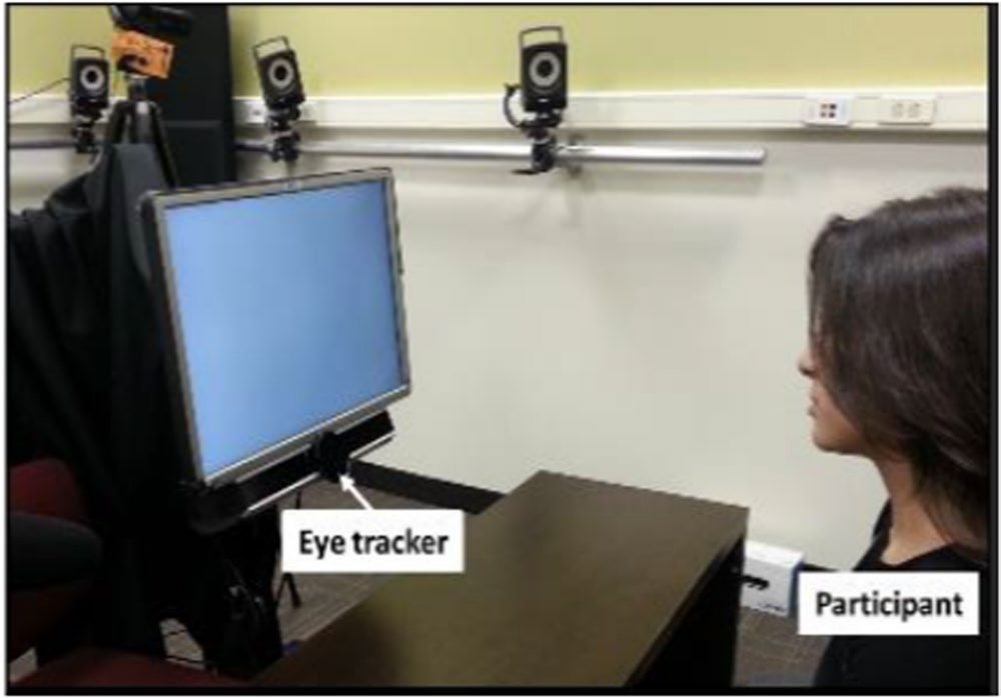
Eyetracker setup for screen-based task.

Participants were seated at a comfortable viewing distance from a computer screen mounted on a movable arm. We explained that they were about to see several adolescents talking about their families, their interests, a vacation they took, and something they don’t like about school. Each of these personal narratives was one minute long and each was presented by a different adolescent, representing different genders and races. We asked participants to passively watch the videos and did not require an overt response. Prior to showing the videos, we performed a five-point calibration of the SensoMotoric Instruments (SMI™) RED eyetracker with a sampling rate of 100 Hz, aiming for <1 degree of deviation in either axis. The RED was attached to a computer monitor mounted on a movable arm, which allowed us to adjust the distance of the tracker and screen to the participant in all directions to obtain optimal recording and viewing parameters (Fig. 4). The calibration screen on the monitor was a white field with a round marker that moved to five pre-determined points on the screen (center and each of the four corners). We asked participants to focus on the marker and the system automatically advanced the marker to the next location after reliably acquiring gaze fixation on that marker location. We repeated calibration until appropriate deviation of x- and y-axes was achieved, at which point we started presentation of the four consecutive narrative videos. Past performance and pilot testing of the task with repeated calibration indicated that there was very little, if any, drift in calibration accuracy over a 20–30 minute period.

### Live interaction task

**Figure 5:**
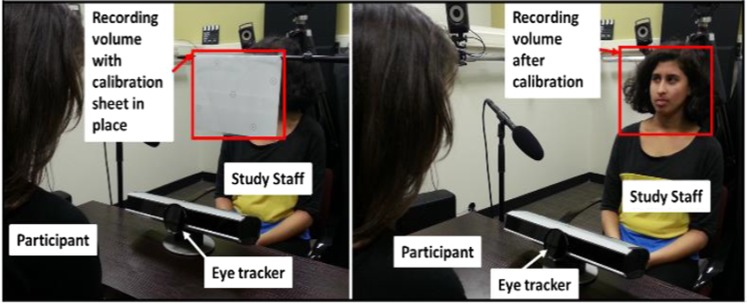
Eyetracker setup for live interaction.

Participants were seated at a small table across from a research assistant, who faced the participant, at a comfortable viewing distance. The setup of table, seating, and viewing distance was the same as for the screen-based task. Given the different heights of participants and RAs, as well as the fact that we did not limit movement with chinrests of other physical constraints, meant that minor shifts in positions of participant, RA, and eyetracker could not be avoided. However, those movements were fairly limited and participants remained within the range of the eyetracker throughout. For the interactive task, we used the movable computer monitor arm to swivel the screen out of the way and the RA was seated in the approximate prior location of the computer screen. We detached the RED eyetracker from the monitor and placed it on the table between the RA and participant, using the RED’s freestanding base, allowing for adjustment of up/down angle, as well as a small riser to adjust height, as necessary. We used Velcro to attach the eye tracker to the table and ensure that it did not shift during the task. We used a laminated white sheet of paper (8.5″ × 11″) as a representation of the recording volume and placed it in front of the conversation partner (Fig. 5). The calibration sheet was attached to an adjustable mic stand and covered the head and neck of the RA to define the spatial volume within which eyetracking would be recorded throughout the live interaction task. Since the height of the participant, the height of their conversation partner, and the precise angle of the eyetracker were different every time, we used a standard tape measure to measure the distances between the calibration sheet and the floor, the calibration sheet and the eyetracker, the participant’s eyes to the floor, and the angle of the eyetracker. We entered those distances into the eyetracking software, allowing the system to process its location in space and interpret the subsequent gaze data. We completed a five-point calibration using the image of the calibration sheet, as recorded by the associated webcam that was recording the RA. The calibration sheet showed black dots in the same locations as during the automated, on-screen version of the calibration. The five markers on the calibration sheet were used in the same sequence as during the on-screen calibration with the same x- and y-axis thresholds to determine adequate calibration. Participants gazed at each of the dots in sequence and the RA made manual adjustments until participant gaze to each of the dots reliably overlapped with the images of the dots on the webcam image. This process established the position of each participant in relation to the calibration volume/RA position as recorded by the webcam. Once calibration was completed, we moved the calibration sheet out of the way and the RA and participant were now able to communicate with each other freely across the small table.

Concurrent with eyetracking data collection, the RED eyetracker recorded frame-synched webcam video and audio of the RA so that each participant’s gaze locations could be superimposed onto video of the recording volume. We also recorded video of the participant using a separate, frame-synched webcam, which allowed us to verify that participants were actively engaged in the task. The webcams were placed in the same locations for all participants. The webcam recording the RA was clamped to the table about a foot to the side of the participant and at approximately the same height as their head. The webcam recording the participant was on a tripod placed three feet behind and to the side of the RA. Both cameras captured the faces and upper torsos of the two conversation partners throughout the interaction. Because the aim of this task was to mimic live social interaction, we positioned all participants the same way at the beginning of the interaction, but did not further restrict the movements of the participants as they were seated in the chair. Over the course of the conversation, participants naturally shifted in their seats, leaned in different directions, and slouched down or sat up straight, similar to the amount of movement available to them during the screen-based task. No participant stood up or drastically changed their position to prevent data capture. The SMI eyetracker has tolerance for participant movements, thereby allowing us to continue recording gaze data throughout the interaction.

The interactive task was modeled after the Double Interview^44^ during which an RA and a research participant interview one another. The interview began when the RA told the participant that they were about to have a conversation in which first the RA would ask questions of the participant that were related to family, vacation, hobbies, and school, and then the roles would be reversed. For the sake of content consistency, these four topics were the same as those used during the videos of personal narratives in the screen-based passive viewing task. During the time that participants interviewed the RA, we provided participants with cues to relevant moments in the RA’s lives if necessary. Cues included photographs of the RA at a family dinner, during a hobby, or on vacation. If photographs were used they were placed vertically within the recording volume and held up only briefly, so participant gaze was not held by the photographs for an extended period of time. Conversations lasted between eight and 20 minutes.

It is important to note that the task demands in the screen-based versus social-interaction paradigms are not directly comparable, since one required active participation and the other only passive viewing. Our primary goal was to investigate whether gaze patterns collected during typical screen-based eyetracking studies can be predictive of gaze patterns in live interactions within participants. We therefore selected a task that represented commonly used “best practices” in screen-based tracking: the stimuli were dynamic video, not static images; the video involved spoken language, providing multi-modal communicative information; the speaker’s face maintained direct gaze on the camera and therefore the participant watching the videos. The live social interaction task we used was designed to mimic realistic conversations, in that we did not manipulate the speaker’s gaze or limit the interaction to a given number of seconds. In both situations, the same participants gazed at a single speaking face in the same lab space, thereby removing between-participant variability and equalizing the environment and visual distractors across both tasks. The cross-task analyses we conduct are not designed to directly compare performance on one task to performance on the other, but rather to determine whether gaze to the face in a passive viewing task can be predictive of that same individual’s gaze to the face during an interactive task.

### Participants

Eighteen adolescents with ASD and 21 NT adolescents were enrolled in this study. The Institutional Review Board of Emerson College approved this study, all experiments were performed in accordance with relevant guidelines and regulations, and we obtained written informed consent from each participant and/or their parents. To ensure that we included only data from participants whose gaze was reliably tracked, we verified the tracking ratio of each participant during the passive viewing task. Tracking ratio indicates the percentage of time over the course of the task that the RED eyetracker successfully tracked the participant’s eyes, regardless of the gaze location. We visually inspected all gaze data to check for flickering, unstable, or intermittent gaze tracking and determined that data with a tracking ratio of less than 70% were not reliable enough to be included. We retained only those participants whose tracking ratio fell above 70%, resulting in a mean tracking ratio of 91% for the ASD group and 95% for the NT group; these ratios did not differ between groups (*t* (32) = 1.65, *p* = 0.11). The final sample included 12 adolescents with ASD (mean age 14:0, 4 females) and 19 NT controls (mean age 12:10, 6 females) with an age range of 10:2 to 17:2.

Participants completed the Core Language Subtests of the Clinical Evaluation of Language Fundamentals, 5th Edition^45^ (CELF-5), the Kaufman Brief Intelligence Test, 2nd Edition^46^ (K-BIT-2), and the Social Communication Questionnaire^47^ (SCQ). The two groups did not differ in age (*F* (1, 30) = 2.76, *p* = 0.11), IQ (*F* (1,30) = 0.57, *p* = 0.46), language ability (*F* (1, 30) = 0.57, *p* = 0.46), or gender (χ^2^ = 0.01, *p* = 0.92, Table 1). ASD diagnosis was confirmed via the ADOS-2^3^ by administrators who achieved research reliability with a certified trainer. The two groups did differ significantly in their scores for the SCQ (*F* (1,30) = 108.26, *p* < 0.0001), showing that individuals in the ASD cohort had significantly more impaired social communication skills than their NT peers, as expected.

**Table 1:** Descriptive Characteristics of both participant groups.

	ASD (n = 12) *M*(*SD*)	NT (n = 19) *M*(*SD*)
Age	14:0 (2:1) Range: 10:5–17:2	12:10 (2:0) Range: 10:2–17:2
Sex	8 male 4 female	13 male 6 female
K-BIT-2	118.67 (19.12) Range: 80–145	114.32 (12.98) Range: 93–147
CELF-5	112.33 (16.66) Range: 89–145	116.74 (15.26) Range: 91–145
SCQ	19.5 (6.29) Range: 12–32	2.68 (2.61) Range: 0–12

## Data Availability

The datasets generated during and/or analyzed during the current study are available from the corresponding author on reasonable request, pending IRB approval of sharing de-identified data.
